# Molecular snapshots of the Pex1/6 AAA+ complex in action

**DOI:** 10.1038/ncomms8331

**Published:** 2015-06-12

**Authors:** Susanne Ciniawsky, Immanuel Grimm, Delia Saffian, Wolfgang Girzalsky, Ralf Erdmann, Petra Wendler

**Affiliations:** 1 Gene Center Munich, Ludwig-Maximilians-Universität München, Feodor-Lynen-Strasse 25, Munich 81377, Germany; 2 Institute of Biochemistry and Pathobiochemistry, Department of Systems Biochemistry, Faculty of Medicine, Ruhr-Universität Bochum, Bochum 44801, Germany

## Abstract

The peroxisomal proteins Pex1 and Pex6 form a heterohexameric type II AAA+ ATPase complex, which fuels essential protein transport across peroxisomal membranes. Mutations in either ATPase in humans can lead to severe peroxisomal disorders and early death. We present an extensive structural and biochemical analysis of the yeast Pex1/6 complex. The heterohexamer forms a trimer of Pex1/6 dimers with a triangular geometry that is atypical for AAA+ complexes. While the C-terminal nucleotide-binding domains (D2) of Pex6 constitute the main ATPase activity of the complex, both D2 harbour essential substrate-binding motifs. ATP hydrolysis results in a pumping motion of the complex, suggesting that Pex1/6 function involves substrate translocation through its central channel. Mutation of the Walker B motif in one D2 domain leads to ATP hydrolysis in the neighbouring domain, giving structural insights into inter-domain communication of these unique heterohexameric AAA+ assemblies.

## Abstract (web summary)

Pex1 and Pex6 form a heterohexameric AAA+ ATPase complex with triangular geometry at the peroxisome membrane. Here the authors use electron microscopy to show that the complex undergoes conformational changes upon ATP hydrolysis, and demonstrate inter-domain communication between neighbouring nucleotide-binding domains.

## Graphical abstract

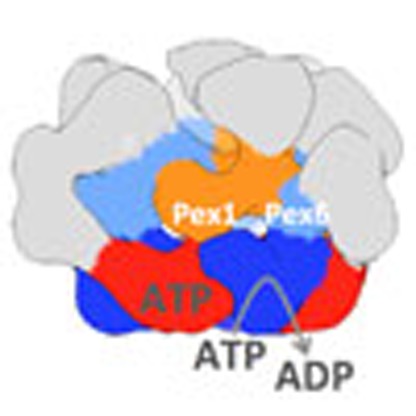

Peroxisomes are self-replicating, single-membrane organelles harbouring enzymes that catalyse important cellular processes such as the detoxification of peroxides and the β-oxidation of fatty acids^1^. The AAA+ (ATPases associated with various cellular activities) proteins Pex1 and Pex6 are essential for peroxisome biogenesis as they are required for the import of folded proteins into the peroxisomal matrix^2^,^3^. At the cytosolic face, both proteins recover the mono-ubiquitinated PTS1 (peroxisomal targeting signal 1) import receptor Pex5 from the peroxisomal membrane to sustain further cycles of protein translocation^4^. In humans, mutations in either the *PEX1* or the *PEX6* gene are the most common cause of severe peroxisomal biogenesis disorders^5^, highlighting the importance of these ATPases in peroxisome function.

Yeast Pex1 and Pex6 form a 700-kDa hexameric complex consisting of stoichiometric amounts of both proteins^6^. They are classified as type II AAA+ ATPases, which by definition contain two conserved nucleotide-binding domains (D1 and D2) in tandem flanked by less conserved N- and C-terminal regions (Fig. 1a). So far, little is known about the nucleotide-dependent dynamics and domain communication of type II AAA+ ATPases, which generally consume energy to exercise mechanical work on their substrate. The ClpA- and ClpB-type of proteins unfold their substrate and thread it through the central channel of a double-tiered hexamer, aided by conserved tyrosine residues in axial pore loops^7^,^8^,^9^. In contrast, structures of type II ATPases NSF (*N*-ethylmaleimide-sensitive factor) and p97 suggest that the energy of nucleotide hydrolysis is transmitted via long-range conformational changes from D1 and D2, respectively, to the *N*-terminal domains, which interact with the substrate^10^,^11^. Hexameric crystal structures of several AAA+ proteins suggest that adenosine triphosphate (ATP) binding and hydrolysis merely moves the axial pore loops^12^,^13^, while cryo EM (electron microscopy) studies of homohexameric AAA+ proteins show that entire ATPase domains undergo nucleotide-dependent movements within one ring^14^ and/or with respect to the second ring^10^,^15^. Most type II AAA+ complexes are homo-oligomers and it has been a major challenge to characterize inter-domain communication between two AAA+ domains in one ring. To our knowledge, the Pex1/6 hexamer is the only type II hetero-oligomeric AAA+ complex allowing for manipulation of only a subset of nucleotide-binding sites and thus dissection of distinct roles of individual subunits concerning complex function, catalytic activity or inter-domain interactions.

Here we present a structural study of nucleotide-dependent movements of the Pex1/6 complex. On the basis of seven different EM maps, we offer an exhaustive structural analysis of the ATPase cycle of Pex1/Pex6 complexes and dissect the structural consequences of ATP binding from those of ATP hydrolysis. Our results show that the domains in the two AAA+ rings undergo profoundly different movements during the ATPase cycle and suggest a mechanism by which the substrate is translocated through the central pore.

## Results

### Pex1/6 hexamers are trimers of dimers

**Figure 1:**
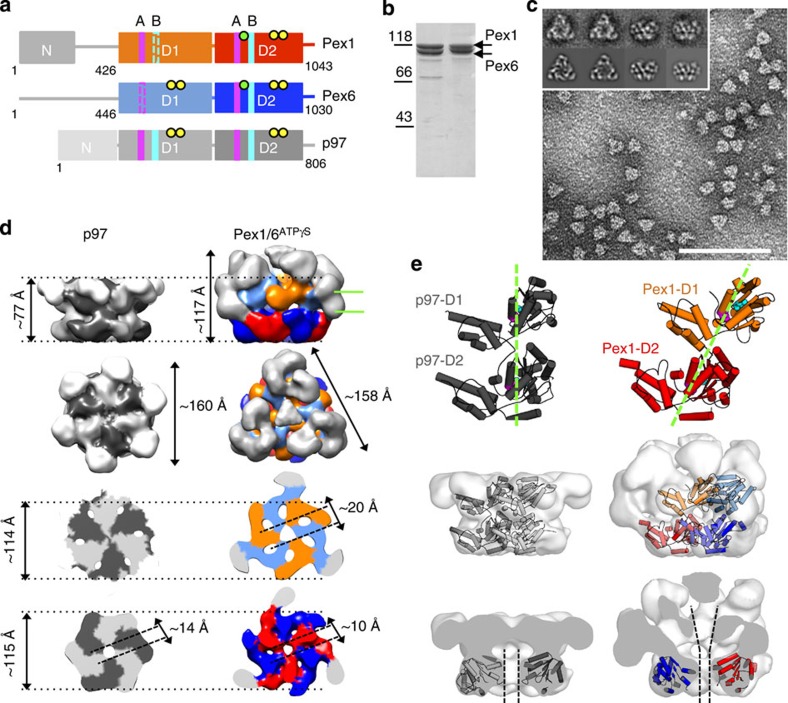
Pex1/6 hexamers are trimers of dimers. (**a**) Schematic domain representation of Pex1/Pex6 protomers compared with p97 (N domain, D1/D2 domain). Conserved motifs and residues of each AAA+ domain are indicated: Walker A (A, magenta bars), Walker B (B, turquoise bars), substrate-binding loops (green dots) and arginine finger residues (yellow dots). Non-canonical Walker A and B motifs are indicated as dotted lines. (**b**) Coomassie-stained SDS–polyacrylamide gel electrophoresis of purified Pex1/6^ATP^ (5 μg, lane 1) or Pex1/6 DWB^ATP^ (5 μg, lane 2) overexpressed in *E. coli* or *Saccharomyces cerevisiae*. (**c**) Raw negative stain electron micrograph showing Pex1/6 complexes (40 μg ml^−1^) incubated with ATPγS. Representative class averages derived from multivariate statistical analysis show top and side views of the Pex1/6^ATPγS^ complex (inset, upper row) and corresponding reprojections of the final 3D reconstruction in the Euler angle directions assigned to the class averages (lower row). Each class contains an average of 5–10 images. Scale bar, 100 nm. (**d**) Pex1/6^ATPγS^ EM density map as side, top and cross-section views of D1 and D2 rings. Colour code: Pex1 D1 (orange), Pex1 D2 (red), Pex6 D1 (pale blue), Pex6 D2 (blue) and Pex1/6N domains (grey). Equivalent views of p97 (pdb-ID: 3CF3) filtered to 20 Å are shown for comparison. p97 single subunits are coloured alternately light and dark grey. Cross-section viewing planes are indicated by green lines. (**e**) Cartoon representation of a p97 protomer without N domains and of a Pex1 protomer homology model, seen from the side of the complex. Domain offset between Pex1 D1 and Pex1 D2 is indicated by green dotted lines. Walker A and Walker B motifs are shown as spheres and coloured as in **a** (upper row). Side view of a p97 dimer fitted as a rigid body into low-pass filtered p97 crystal structure and Pex1/6 heterodimer docked to Pex1/6^ATPγS^ 3D map (middle row). Cut-open side views of the low-pass filtered p97 crystal structure with p97 D2 placed into the EM density map and of Pex1/6^ATPγS^ map with fitted Pex1 D2 and Pex6 D2 homology models. Black dotted lines indicate the central channel (lower row).

The D2 domains of both peroxins Pex1 and Pex6 are strongly conserved, displaying Walker A and B motifs, essential for ATP binding and hydrolysis, respectively, and two spaced out arginine fingers, which are generally thought to sustain oligomerization and to participate in ATP hydrolysis at protomer interfaces^16^. Besides, in the D2 domain of both peroxins a conserved aromatic residue is located in the substrate-binding loop region (Fig. 1a; Supplementary Fig. 1). ATP binding to the D2 domains was shown to be essential for complex formation and peroxisome biogenesis^17^. The D1 domains on the other hand are poorly conserved without a canonical Walker B (WB) motif, arginine fingers or conserved aromatic residues in the pore loop in Pex1 D1. The Pex6 D1 domains harbour two non-conserved arginine residues in the region where arginine fingers are expected (Supplementary Fig. 1), and contain a reduced Walker A motif sufficient for either adenosine diphosphate (ADP) or ATP binding^18^ but lack the WB motif.

To resolve the hexameric structure of the Pex1/6 complex, we examined the negatively stained assembly by EM and single-particle image analysis techniques. The proteins were purified from either yeast or *Escherichia coli* overexpression systems^6^ and assembled in the presence of a nucleotide (Fig. 1b). As sequence similarity searches show, Cdc48/p97 is the closest type II AAA+ homologue of yeast Pex1 and Pex6. The overall shape of the complex was thus expected to resemble the hexameric structure of p97. Surprisingly, the complex adopts a trimeric symmetry resembling an equilateral triangle when viewed from the top and featuring a clear double layer capped by additional density when viewed from the side (Fig. 1c). We established the domain allocation in the double layer by examining Pex1GST/Pex6 complexes in the presence of ATPγS by negative stain EM. Two-dimensional side-view class averages show additional density attributed to the C-terminal glutathione *S*-transferase (GST) moiety emerging from the lower tier of the Pex1/6 complex (Supplementary Fig. 2a). The D1 and D2 domains of Pex1/6 thus form the top and bottom tier of the double layer, respectively.

In the three-dimensional (3D) reconstruction of negatively stained Pex1/6 complexes assembled in the presence of the slowly hydrolysable ATP analogue ATPγS (Pex1/6^ATPγS^) at 21 Å resolution, the two AAA+ rings form the core of the complex and display a distinct sixfold symmetry, although a threefold symmetry was used for reconstruction (Fig. 1d; Supplementary Fig. 2b). The height and width of the double tier resembles that of p97. While the N termini of p97 reside next to the D1 domains, the N termini of Pex1 and Pex6 constitute the top layer and the vertices of the triangular structure. The asymmetric arrangement of the N termini, which so far has not been observed for other AAA+ ATPases, gives rise to the characteristic triangular shape of the heterohexameric complex and indicates an alternating order of Pex1 and Pex6 in the hexamer. A comparison between the Pex1/6^ATPγS^ complex and the structure of Δ188Pex1/Pex6^ATPγS^ hexamers assigns missing density in every second subunit of the Δ188Pex1/Pex6 ^ATPγS^ complex to the N terminus of Pex1 (Supplementary Fig. 2c). Thus, the vertices of the triangular structure are mostly made up by the Pex6 N termini. We also created truncated Pex1/6 complexes, missing up to 400 amino acids at the Pex6 N terminus. However, Pex6 N-terminal deletions result in unstable protein expression or compromised hexamerization.

The tight and relatively static assembly of the D1 ring in p97 lead to the suggestion that p97 unlike ClpB does not thread its substrates through the central channel of the hexamer^19^. In contrast, the Pex1/6^ATPγS^ hexamer forms a central channel throughout the double tier, which is even wider in the D1 ring than in the D2 ring (Fig. 1d,e). The D2 ring of a hexameric crystal structure of p97 (pdb-ID: 3CF3 (ref. 11) can be fitted as a rigid body into the D2 ring of our Pex1/6^ATPγS^ EM map (Supplementary Fig. 2d), confirming that the Pex1/6 structure is not significantly altered by the stain. Next, we fitted homology models of Pex1 or Pex6 D1 and D2 ATPase domains as rigid bodies into the EM density using the automatic fitting procedure in UCSF Chimera. All starting positions tested resulted in the same position of the models in the EM map, indicating only one local minimum for each fit (Supplementary Fig. 2d). In contrast to the p97 D1 and D2 rings, which are located on top of each other in hexameric crystal structures^20^,^21^, the Pex1/6^ATPγS^ ATPase rings are offset by ∼30° when viewed from the top (Fig. 1e). Therefore, the AAA+ double layer in Pex1/6^ATPγS^ differs from the one in p97 by an offset between the D1 and D2 domains and a more expanded D1 ring to account for a 20-Å central pore in this layer.

### D2 domains rotate downwards upon ATP hydrolysis

**Figure 2:**
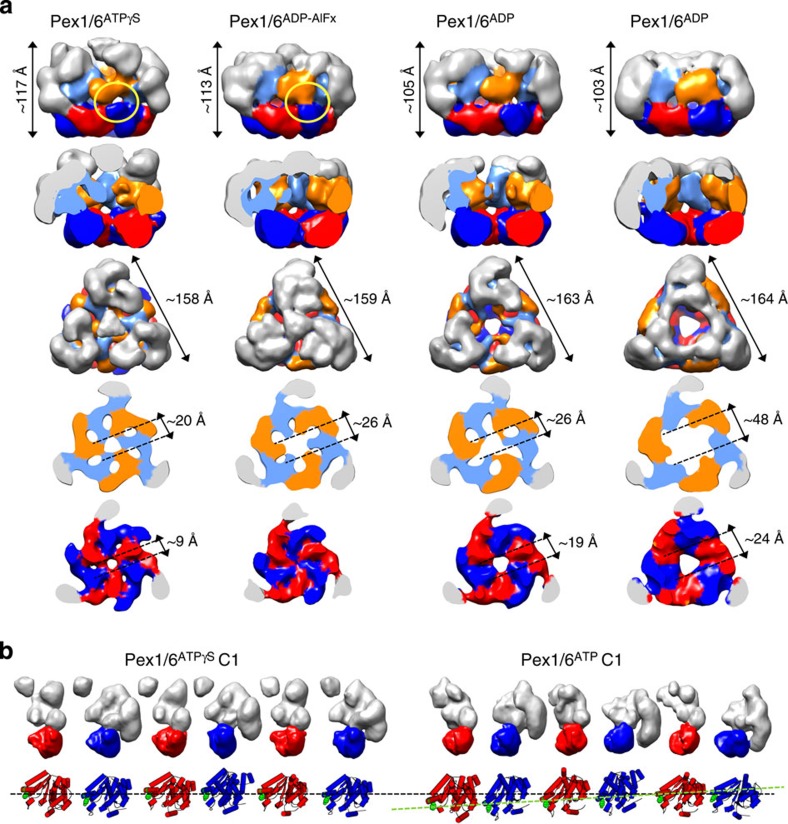
Symmetric and asymmetric wild-type Pex1/6 complexes in the presence of different nucleotides. (**a**) EM reconstructions of Pex1/6 complexes in the presence of ATPγS (Pex1/6^ATPγS^), ADP-AlFx (Pex1/6^ADP-AlFx^), ATP (Pex1/6^ATP^) and ADP (Pex1/6^ADP^) seen as side views (upper row), followed by cut-open side views, top views and cross-sections of the D1 and D2 layers (lower rows). Domain colours correspond to Fig. 1d. (**b**) Surface representation of each protomer of the asymmetric Pex1/6^ATPγS^ and Pex1/6^ATP^ complex. Pex1 (red) or Pex6 (blue) D2 domains are highlighted. Underneath, a cartoon representation based on rigid body fits of homology models into negative stain EM maps is shown. Pex1^F771^ and Pex6^Y805^ are shown as green spheres. The black dotted line indicates the position of pore-facing loops in the D2 domains of asymmetric Pex1/6^ATPγS^. A green dotted line indicates the asymmetric arrangement of D2 domains in Pex1/6^ATP^.

To investigate the effect of different nucleotides on the hexameric arrangement, we generated 3D reconstructions of Pex1/6 complexes in the presence of the transition state analogue ADP-AlFx, ATP or ADP (Fig. 2a). Refinement yielded maps with 21–24 Å resolution and good agreement between input class averages and corresponding reprojections (Supplementary Figs 2b and 3a–c). Refinement of our ADP-AlFx and ATPγS structures from inter-changed starting models yields the original structures (Supplementary Fig. 4a–f).

For all EM maps, the triangular complex shows a similar overall height (103–117 Å) and edge length (158–164 Å). The height of the Pex1/6 AAA+ double tier remains almost identical in the presence of different nucleotides (75–78 Å) and all AAA+ rings display the previously observed sixfold symmetry. Notably, in the presence of ADP, Pex1/6 complexes display the largest central pore in the D2 domain and least defined domain appearance when compared with all other nucleotide states. Thus, addition of ATP or its analogues notably structures the D2 ring, indicating nucleotide binding to this AAA+ layer. This is in agreement with data showing that ATP binding to the D2 ring, but not to the D1 ring, is essential for peroxisome biogenesis^17^,^18^. The differences between the Pex1/6^ATP^ and Pex1/6^ATPγS^ complexes are small, accounting only for ∼5–10-Å-wider central pores in the D1 and D2 rings. To examine each domain position individually, we refined the maps without imposing symmetry and fitted the D2 domains using Chimera (Supplementary Figs 4g,h and 5a). While the D2 domains form a planar ring in the presence of ATPγS, they adopt an asymmetric arrangement in the presence of ATP (Fig. 2b). This observation indicates that in the presence of ATP, the D2 domains are hydrolysing or not fully occupied with nucleotides. We conclude that prolonging nucleotide binding to the ATP-binding pocket by a slowly hydrolysable analogue such as ATPγS, promotes a crystal structure-like packing of the D2 domains that allows for arginine finger contacts and ATP hydrolysis.

**Figure 3:**
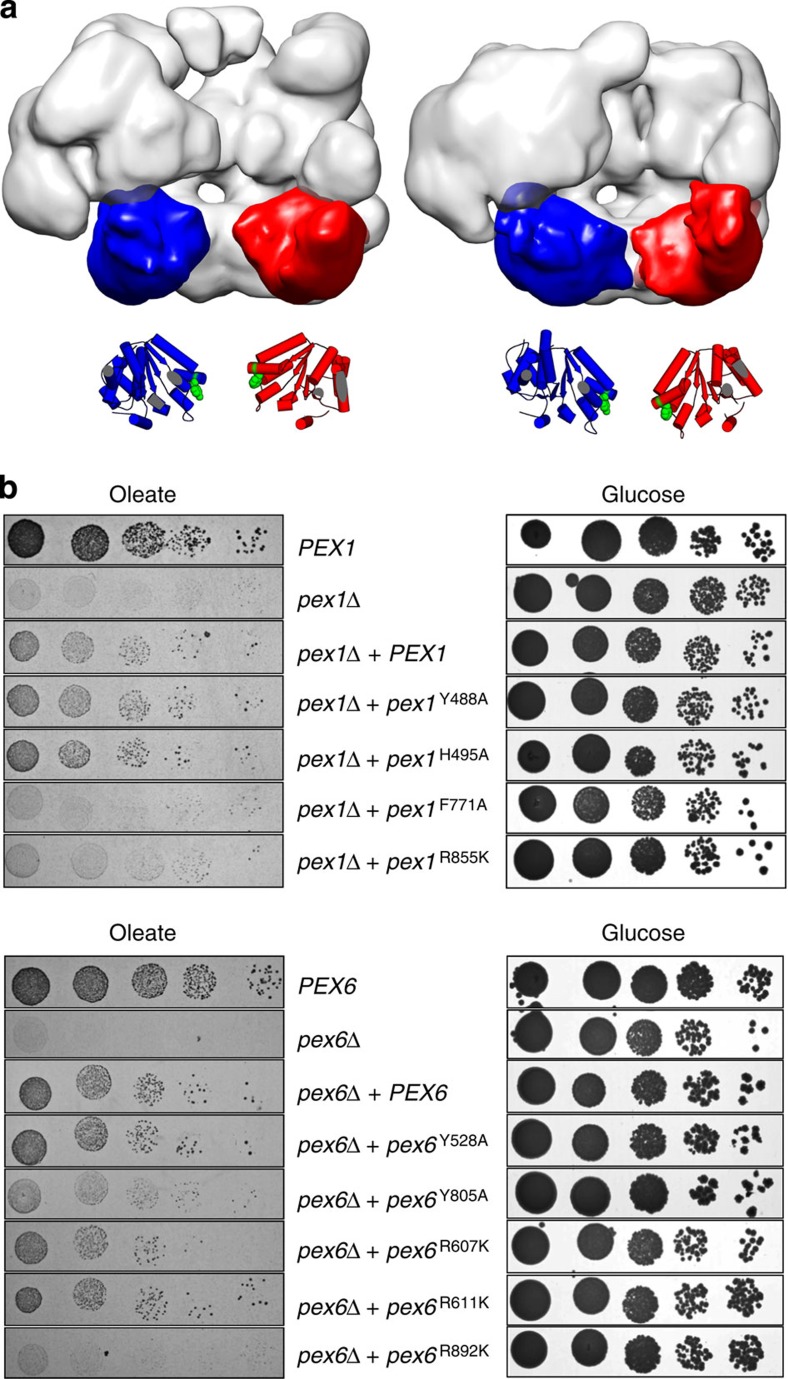
ATP hydrolysis translocates tyrosine loops through movements of D2 domains. (**a**) Side-view surface representation of Pex1/6^ATPγS^ and Pex1/6^ADP-AlFx^. One heterodimer is omitted from the hexamer. One Pex1 D2 and Pex6 D2 domain of each complex is highlighted in red and blue, respectively. Underneath, a cartoon representation based on rigid body fits of homology models into negative stain EM maps is shown. Conserved aromatic residues Pex1^F771^ and Pex6^Y805^ are shown as green spheres. (**b**) Growth of strains expressing either wild-type (*PEX1*, *PEX6*), no (*pex1Δ*, *pex6Δ*) or mutated *pex1*, *pex6* alleles with a modified pore loop (*pex1*^Y488A^, *pex1*^H495A^, *pex1*^F771A^, *pex6*^Y528A^, *pex*6^Y805A^) or arginine finger residues (*pex1*^R855K^, *pex6*^R607K^, *pex6*^R611K^, *pex6*^R892K^) on either glucose or oleate as a single carbon source.

In the presence of ADP-AlFx, AAA+ domains are trapped in a transition state between ATP and ADP^22^. In comparison with the ATPγS-bound complex, the pore in the Pex1/6^ADP-AlFx^ D1 ring is slightly wider and the pore in the D2 ring is closed (Fig. 2a). Pore closure in D2 is accompanied by an anticlockwise rotation of the AAA+ domains and appearance of strong connecting densities between the Pex6 D2 domains and the Pex1 D1 domains on the outside of the complex (Fig. 2a, yellow circle). This inter-protein contact is only possible because of the offset between the D1 and D2 domains. Furthermore, the Pex1 N termini on top of the complex close the central opening on the symmetry axis, while the lateral N-terminal Pex6 densities remain roughly in place between both states. In analogy to the fit into Pex1/6^ATPγS^, we automatically docked the D2 homology models into the Pex1/6^ADP-AlFx^ EM density (Supplementary Fig. 5a). When we interpret the domain rotations on the basis of the fits, it is obvious that the pore-facing elements of the D2 domains are rotated down towards the C-terminal exit of the central pore that crosses both AAA+ rings (Fig. 3a). From related AAA+ hexamers ClpA, ClpX and p97, it is known that substrate-binding motifs in the D2 domains are required for efficient substrate remodelling^7^,^19^,^23^. Thus, we were interested to know whether conserved aromatic residues in potential substrate-binding motifs of Pex1 or Pex6 are essential for complex function (Supplementary Fig. 1). We therefore tested the impact of the respective point mutations in oleate growth assays (Fig. 3b). Point mutations of neither Pex1 nor Pex6 D1 (Pex1^Y488A^, Pex1^H495A^ and Pex6^Y528A^) show any growth effect using oleate as a sole carbon source. Strikingly, D2 domain mutants Pex1^F771A^ and Pex6^Y805A^ were both unable to metabolize oleate, indicating that peroxisomal biogenesis depends on full or limited substrate threading through the central pore of the Pex1/6 complex. It also suggests that the D2 domains of both proteins interact with the substrate. Pex1^F771^ and Pex6^Y805^, both located in the D2 substrate-binding loop, are facing the central pore when AAA+ domains of Pex1 and Pex6 homology models are automatically fitted as rigid bodies to our EM maps (Supplementary Fig. 5b). Furthermore, the residues are displaced by ∼10 Å through movements of the entire AAA+ domain between the ATPγS-bound and ADP-AlFx-bound state of the Pex1/6 complex (Fig. 3a).

### Pex6 D2 accounts for main ATPase activity of the complex

**Figure 4:**
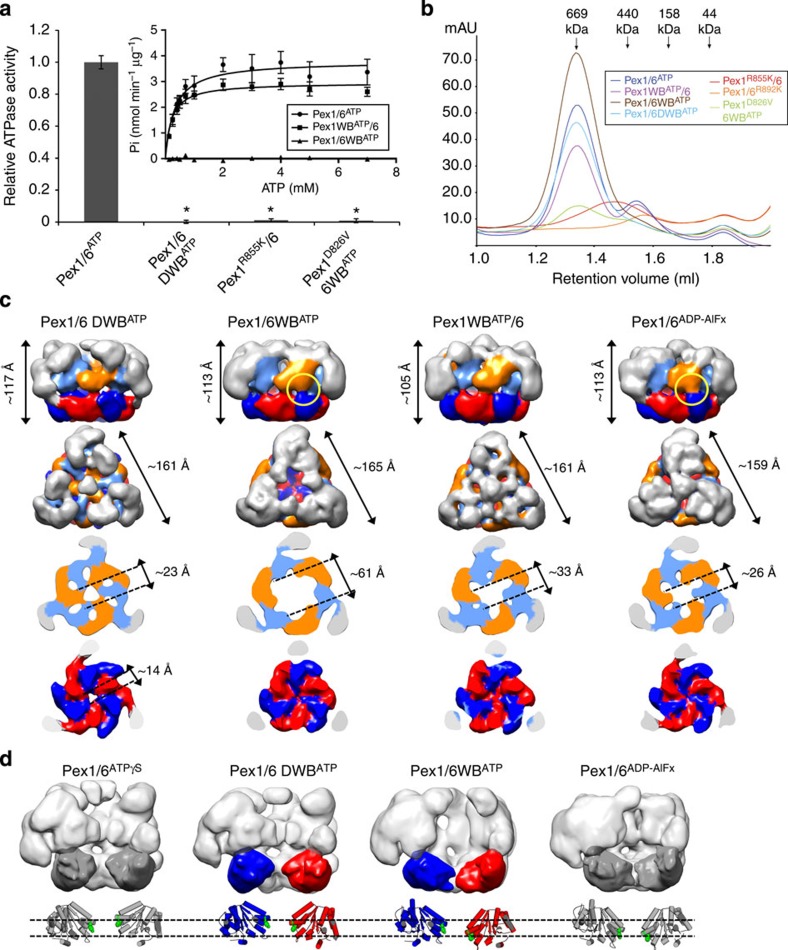
ATPase activity of Pex6 D2 domains drive conformational changes. (**a**) ATPase activities of wild-type Pex1/6 (Pex1/6^ATP^), single Walker B (Pex1WB^ATP^/6, Pex1/6WB^ATP^) or double-Walker B mutants (Pex1/6 DWB^ATP^) or Pex1 arginine finger (Pex1^R855K^/6) or ISS motif (Pex1^D826V^/6WB^ATP^) mutants. Assays were performed in the presence of 1 mM ATP and 5 mM Mg^2+^ at 37 °C. Error bars represent s.d. of two independent experiments with five technical replicates each. *P<0.0001 in comparison with wild-type Pex1/6^ATP^ (one-way ANOVA). Michaelis–Menten plot of enzyme activity of Pex1/6^ATP^, Pex1WB^ATP^/6 or Pex1/6WB^ATP^ at various ATP concentrations (inset). Error bars represent s.d. of two independent experiments including three technical replicates. (**b**) Size-exclusion chromatography A_290 nm_ profiles of purified Pex1/6 complexes used for ATPase activity assays. (**c**) EM reconstructions of double- (Pex1/6 DWB^ATP^) or single- (Pex1/6WB^ATP^, Pex1WB^ATP^/6) Walker B complexes in the presence of ATP as side views (upper row), followed by top views and cross-sections of the D1 and D2 rings (lower rows). (**d**) Side-view surface representation as in Fig. 3a. Underneath, a cartoon representation based on rigid body fits of homology models into negative stain EM maps is shown. Pex1/6^ATPγS^ and Pex1/6^ADP-AlFx^ are depicted in grey for comparison. Domains are coloured according to Fig. 1d. Green spheres depict residues Pex1^F771^ and Pex6^Y805^.

Our previous work already established that the isolated wild-type complex exhibits a basal ATPase activity comparable to p97 (ref. 6). To determine whether both D2 domains contribute equally to overall ATPase activity, we measured the ATPase activities of mutant complexes carrying a WB mutation either in the Pex1 D2 domain (Pex1WB^ATP^/6), in the Pex6 D2 domain (Pex1/6WB^ATP^) or both domains Pex1/6 DWB^ATP^ (Fig. 4a). In all cases, the conserved glutamate of the WB motif was exchanged for glutamine, which typically inhibits nucleotide hydrolysis but not its binding^16^. Compared with wild-type levels, the ATPase activity is slightly reduced when Pex1 D2 carries a WB mutation (Fig. 4a, inset) and abolished in the Pex1/6WB^ATP^ and Pex1/6 DWB^ATP^ complex. Since strains carrying a Pex1WB^ATP^ mutation almost show wild-type growth using oleate as a sole carbon source, we can rule out any essential hydrolytic activity in the Pex1 D2 domain (Supplementary Fig. 5c). Intriguingly, both D2 domains contain conserved arginine fingers (Supplementary Fig. 1), and mutation of the arginine to lysine eliminates growth on oleate (Fig. 3b) hinting that both domains are able to hydrolyse ATP. Work on the heterohexameric type I ATPase Yta10/Yta12 suggested that the arginine finger and residues in the inter-subunit signalling (ISS) motif mediate communication between subunits^24^. To test whether we can uncouple potential communication between the Pex1 and Pex6 D2s and unleash Pex1 ATPase activity, we mutated the Pex1 arginine finger (Pex1^R855K^/6) or a conserved aspartic-acid residue in the ISS motif in the Pex1/6WB^ATP^ complex (Pex1^D826V^/6WB^ATP^). Still, the complex ATPase activity is negligible, suggesting that Pex1 D2 has only a very low intrinsic hydrolytic activity. Moreover, negative stain EM of Pex1^R855K^/6 complexes (Supplementary Fig. 3h) and size-exclusion chromatography profiles of both arginine finger mutants (Fig. 4b) imply that mutation of either arginine finger impairs complex formation and causes the growth defect on oleate. Thus, the conserved D2 arginine fingers Pex1^R855K^ and Pex6^R892K^ are essential for complex formation.

### WB mutation induces hydrolysis in the adjacent AAA+ domain

Finally, to structurally dissect the influence of nucleotide binding to distinct binding pockets, we generated 3D reconstructions of all complexes carrying WB mutations (Fig. 4c; Supplementary Fig. 3e–g). The overall shape of the complex is not altered by the mutations. In fact, the structures of Pex1/6 DWB^ATP^ and Pex1/6^ATPγS^ are almost identical, supporting the notion that prolonged ATP binding to the nucleotide-binding pockets creates a packing of the D2 domains in the EM structure that resembles that of hexameric p97 crystal structures. The similarity between these independently generated 3D reconstructions further corroborates the structural integrity of the complexes during negative staining and highlights the consistency of our image-processing procedures.

The reconstructions of the Pex1/6WB^ATP^ and Pex1WB^ATP^/6 complexes resemble the Pex1/6^ADP-AlFx^ complex in the orientation of the D1 domains and in the arrangement of the N termini. Similar to the Pex1/6^ADP-AlFx^ complex, the pore in the D2 ring is closed in both WB reconstructions. The Pex1/6WB^ATP^ reconstruction also shows a strong contact between Pex6 D2 domains and Pex1 D1 domains, suggesting that Pex1 adopts a transition state orientation, when Pex6 D2 is permanently bound to ATP (Fig. 4c, yellow circle). The D1 layer of both complexes forms a significantly wider cavity than Pex1/6^ADP-AlFx^, extending up to 33 and 61 Å in diameter for Pex1WB^ATP^/6 and Pex1/6WB^ATP^, respectively. Particularly the D1 domains of the hydrolytically inactive Pex1/6WB^ATP^ complex adopt an atypical AAA+ arrangement, strongly suggesting that neighbouring AAA+ domains cannot be firmly connected via rigid body interactions as observed in single-ring AAA+ proteins such as ClpX or the 19S proteasomal ATPases^25^,^26^. Although the hydrolytically active Pex6 D2 domain is trapped in the ATP-bound state in both the Pex1/6 DWB^ATP^ and the Pex1/6WB^ATP^ complex, their overall 3D structures differ significantly. Closer examination of the D2 layer in the Pex1/6WB^ATP^ and docking of homology models to our EM maps shows a staggered arrangement of the D2 domains within the threefold symmetry unit (Fig. 4d; Supplementary Fig. 5a,b). The wild-type Pex1 domain adopts a transition state-like orientation indicating that permanent binding of ATP to Pex6 D2 causes Pex1 D2 to hydrolyse ATP. Since the complex does not exhibit significant ATPase activity, we conclude that after hydrolysis nucleotide exchange in Pex1 D2 is impaired causing the domain to only hydrolyse one ATP before the complex becomes trapped.

## Discussion

We provide a comprehensive structural and biochemical study of a heterohexameric type II AAA+ complex in action. The overall domain assignment and ATPase activity of the Pex1/6 complex have been reported very recently^27^, but our analysis substantially extends the current findings with regard to the number of resolved steps in the ATPase cycle and *in vivo* data in support of our findings. Dimers consisting of Pex1 and Pex6 assemble into a trimer with an atypical triangular geometry. The unusual shape of the complex is constituted by the laterally positioned Pex6 N termini on every second protomer in the hexamer (27, our data). While the location of the Pex6 N termini is barely altered during ATP hydrolysis, the N termini of Pex1 move in a nucleotide-dependent fashion between ATP-bound and ADP-AlFx states. It has been proposed that the N-terminal double-ψ-barrel-fold domain of Pex1 binds adaptors or substrates just like its homologues NSF or p97 (ref. 28). Hence, mobility of Pex1N might reflect interactions with substrate and/or adaptor proteins. On the other hand, Pex6 N termini are thought to locate the ATPase complex to the peroxisomal membrane through binding to Pex15 (ref. 18). The fixed arrangement of Pex6N in all nucleotide states likely reflects a physiological conformation, placing the complex to the peroxisomal membrane via interactions with Pex15. In addition, since hexamerization is compromised in Pex6 N-terminal deletion mutants, peripheral Pex6 N domains sustain the hexameric state of Pex1/6 complexes.

In our hands, the ATPase activity of the Pex1/6 complex is attributed solely to the Pex6 D2 domains. In accordance with the findings by Gardner^27^, the D1 and Pex1 D2 domains of the isolated complex have barely any ATPase activity, while the relatively weak basal activity of Pex6 D2 is in line with data from other AAA+ family members^6^,^27^. However, while it was previously suggested that Pex1 hydrolysis is inhibited by Pex6 (ref. 27), our *in vivo* studies clearly show that Pex1 D2 ATP hydrolysis is dispensable for complex function. It is plausible that Pex1 activity is necessary under certain growth conditions and that it can be stimulated in the presence of substrate or co-factors. Similar dependencies were found for HslU and Yta10/12 complexes, suggesting a regulatory link between substrate-binding loops and the ATP-binding pocket^4^,^24^,^29^. In addition, Pex1 ATPase activity could be regulated by contacts between the Pex1 D2 alpha helical domain and the Pex6 N terminus, which we observe in all ATP-bound wild-type complexes, but which might be altered upon Pex6N binding to Pex15.

**Figure 5:**
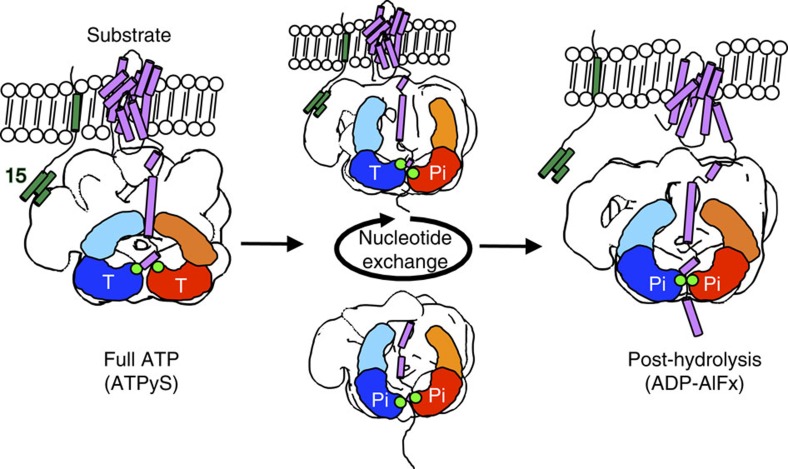
Model for Pex1/6 movements during ATP binding and hydrolysis. The Pex1/6 complex anchors to the peroxisomal membrane via binding of Pex6 N domains to Pex15. Pex1 N domains establish interactions with the substrate. ATP binding to Pex1 D2 and Pex6 D2 (full ATP, ATPγS) elevates substrate-binding loops in the D2 domain, ready to grab the substrate. ATP turnover creates a power stroke that pulls the substrate along the central pore (post hydrolysis, ADP-AlFx). Nucleotide exchange in Pex6 D2 or Pex1 D2 translocates the substrate along the central pore (Pex1/6WB^ATP^, Pex1WB^ATP^/6). One Pex1 and Pex6 protomer are denoted as a simple cartoon representation. Conserved aromatic residues of substrate-binding loops are shown as green dots. Representative tertiary structures of substrate protein (purple) and membrane anchor Pex15 (green) are depicted as cartoon representations. Nucleotide occupancy of each D2 domain is indicated by T for ATP or Pi for the transition state.

Our results suggest that both D2 domains of the Pex1/6 complex interact with the substrate, as they harbour conserved aromatic residues in their substrate-binding loops that are essential for complex function *in vivo*. Docking of homology models to our EM maps reveals a pore-facing localization of the Pex1/6 substrate-binding loops in all examined nucleotide states, which is consistent with the location of substrate-binding loops in hexameric crystal structures of related AAA+ proteins^30^,^31^,^32^,^33^. In particular, domain rotations observed between the Pex1/6^ATPγS^ and ADP-AlFx states reflect a power stroke that pulls the substrate towards the C-terminal exit of the central tunnel upon ATP hydrolysis. Although a substrate threading has not been shown for Pex1/6 yet, our data strongly support such mechanism. A wealth of biochemical studies shows that AAA+ proteins bind the substrate when occupied with ATP but not ADP^34^,^35^,^36^. In the presence of ATP or its analogues, the substrate-binding motifs of Pex1/6 are most elevated and almost reach the centre of the complex while they are positioned close to the C-terminal rim of the central pore when ADP-AlFx is present (Fig. 5). In contrast to the tightly closed D1 layer seen in hexameric crystal structures of p97/Cdc48 (ref. 21), the D1 domains of Pex1/6 complexes are highly mobile and maintain a central pore open to threading in all nucleotide states. Furthermore, the domain architecture of Pex1/6 resembles Hsp100/Clp type AAA+ proteins, which all translocate their substrate through a central channel, more than it resembles p97/Cdc48 and NSF, which have been proposed to remodel substrate by alternative models^10^,^21^. However, recent EM studies also suggest a substrate translocation mechanism for p97/Cdc48 complexes^37^. Left without ATPase activity or conserved substrate-binding loops, the role of the D1 domains remains enigmatic. A conformational flexibility of the D1 layer was also observed in EM reconstructions of Hsp104 and p97 (refs 14, 38) hinting at a conserved function. We speculate that the D1 domains might fulfil structural tasks during ATP hydrolysis in D2, such as (a) transmission of small movements in D2 into larger N-terminal motion, (b) stretching and thus processing of the substrate by domain rotation and closure of the central channel at the N-terminal domains and the D2 domains (as seen for the ADP-AlFx state) or (c) pushing the substrate towards the central pore of the D2 ring.

All in all, we observe two types of movement in the D2 ring: one out-of-plane rotation of the D2 domains that moves the AAA+ domains along the symmetry axis and an in-plane rotation of the D2 domains that opens and closes the D2 pore. The latter movement, albeit less pronounced (up to 24 Å opening), has been observed in hexameric crystal structures of HslU, the large tumour antigen helicase, dynein and ClpX^13^,^26^,^39^,^40^, and in all cases it was caused by hinge movements between the large and the small AAA+ subdomains. Similar to our observations, the pore in the crystal structures is small when ATP is bound and wide when ADP or no nucleotide is bound. Intriguingly, an out-of-plane rotation of the entire AAA+ domain in the transition state has also been observed for hexameric crystal structures of p97 (ref. 12). Here, the D2 domains are rotated away from the D1 domains by 9° in the presence of ADP-AlFx in comparison with the ATP-bound state, causing a translocation of the substrate-binding loops towards the C-terminal exit of the central channel by 10 Å. Our maps show a qualitatively and quantitatively similar movement. They suggest that large parts of the D2 domain rotate upon ATP hydrolysis thereby translocating the substrate.

Our EM maps suggest that prolonged ATP binding to the Pex1/6 D2 domains results in a planar, hexameric arrangement in this layer akin to hexameric crystal structures of p97, while nucleotide hydrolysis causes a downward rotation of the D2 domains (Fig. 5). If one of the D2 subunits in the heterohexamer is bound to ATP, the neighbouring domain adopts a transition state-like orientation, as seen in complexes carrying a single Walker mutation. We conclude that for ATP hydrolysis to occur, at least two neighbouring domains need to be ATP bound. Only when the left-hand domain is arranged so that interacting motifs such as the arginine finger are positioned correctly, the right-hand domain is able to hydrolyse ATP (full ATP, Fig. 5). Upon ATP hydrolysis, the D2 domains translocate downwards along the pore, presumably pulling the bound substrate along (post hydrolysis, Fig. 5). Thus, repeated ATP hydrolysis in Pex6 D2 would structurally be represented by alternation between the 1WB^ATP^/6 and Pex1/6 DWB^ATP^ conformation of the complex, generating a downward pull in the D2 domains (Supplementary Movie 1). The detailed and comparative snapshots of the Pex1/6 complex during its ATPase cycle let us propose a model, whereby the hexamers thread their substrate protein(s) by action of the D2 domains through the central channel to unfold it either partially or completely. They also shed light onto the complex dynamics of the N-terminal and D1 domains, whose exact functions remain to be elucidated.

## Methods

### Plasmid construction and mutagenesis

Full-length yeast *PEX1* and *PEX6* were amplified from genomic DNA by PCR. The N-terminal truncation mutant Δ188*PEX1* was produced by PCR amplification of a gene segment corresponding to amino acids 189–1,043. Resulting constructs, together with a C-terminal, TEV (tobacco etch virus)-cleavable protein A (ProA)-tag, were cloned into HindIII/BamHI and KpnI/BamHI sites of plasmids pRS425GAL and pYES2. Plasmids used in oleate growth assays were generated through PCR amplification of *PEX1* and *PEX6* open-reading frames from genomic DNA comprising 5′ flanking regions. Resulting fragments were inserted into SacI/XhoI and XbaI/KpnI sites of plasmid pRS316. For *E. coli* expression of HisPex1GST and HisPex6, yeast *PEX1* and *PEX6* obtained from PCR were cloned into the SacI/HindIII site of pRSFDuet-cGST and the BamHI/SalI site of pRSFDuet-1 (Merck). pRSFDuet-cGST was obtained by cloning the PCR product of *GST* including the thrombin cleavage site, with pGEX-4T-2 (GE Healthcare) as template, into HindIII/NotI site of pRSFDuet-1 (Merck). Point mutations were introduced using the Quick Change site-directed mutagenesis method (Stratagene). All plasmids are listed in Supplementary Table 1 and PCR primers are listed in Supplementary Table 2.

### Protein purification

Recombinant proteins were overexpressed at 28 °C for 16 h and induced with 2% galactose (Supplementary Table 3). The harvested cells were resuspended in Buffer A1 (20 mM HEPES pH 7.5, 100 mM NaCl, 10 mM MgCl_2_, 10% glycerol, 2 mM ATP and 0.5 mM dithiothreitol (DTT)) containing 20 U DNaseI, 1 mM phenylmethylsulphonyl fluoride, one complete EDTA-free mini protease inhibitor cocktail tablet (Roche) and were lysed using glass beads. The lysate was cleared by centrifugation (4 °C, 30 min, 25,099*g*) and the supernatant was applied on a gravity flow column packed with IgG Sepharose 6 Fast flow beads (GE Healthcare). The beads were washed with a 15-fold matrix volume of buffer A1, 15-fold matrix volume of Buffer A1 containing 500 mM NaCl followed by 15-fold matrix volumes of Buffer A1. TEV cleavage of immobilized proteins was performed for 80 min at 20 °C in Buffer A1. Eluted complexes were separated from contaminants and TEV protease by centrifugation (SW40 rotor, 285,000*g*, 16 h, 4 °C) through a 10–40% glycerol gradient in Buffer A2 (20 mM HEPES pH 7.5, 100 mM NaCl, 10 mM MgCl_2_, 2 mM ATP and 0.5 mM DTT). Separated proteins were fractionated and analysed by SDS–polyacrylamide gel electrophoresis (Supplementary Fig. 5d).

Expression of recombinant proteins in *E. coli* Tuner (DE3) cells (Merck) was performed at 20 °C in the presence of 0.4 mM isopropylthiogalactoside for 20 h (HisPex1GST) or 5 h (HisPex6). Cells were harvested, resuspended and combined in AAA-buffer I (50 mM Tris, 300 mM NaCl, 5 mM MgCl_2_, 5 mM ATP, 1 mM DTT and 40 mM imidazole, pH 7.4) containing selected protease inhibitors (1 mM phenylmethylsulphonyl fluoride, 8 mM anitpain, 0.3 mM aprotinin, 0.16 mg ml^−1^ benzamidin, 1 mM bestatin, 10 mM chymostatin, 5 mM leupeptin and 15 mM pepstatin) and homogenized using an EmulsiFlex-C5 (Avestin). The 45,000 *g* supernatant was incubated for 2 h with Ni^2+^-NTA-Agarose (5PRIME). Agarose was transferred into centrifuge columns (Thermo) and washed with 20-fold matrix volume of AAA-buffer I. Bound proteins were eluted in two steps with AAA-buffer II (50 mM Tris, 300 mM NaCl, 5 mM MgCl_2_, 5 mM ATP and 10 mM DTT, pH 7.4) containing 100 and 300 mM imidazole, respectively. Further purification was achieved by incubation of the Ni^2+^-NTA eluate with Glutathione Agarose (Macherey-Nagel) for 1 h and washing of the agarose with sixfold matrix volume AAA-buffer II containing 200 mM imidazole using centrifuge columns (MoBiTec). Pex1/6 complexes were eluted in washing buffer by overnight thrombin cleavage at 4 °C and final centrifugation.

### ATPase assays

Pex1/6 complexes were applied on a Superose6 PC 3.2/30 column, equilibrated with 50 mM Tris, 300 mM NaCl, 200 mM imidazole, 10 mM DTT and 2 mM ATP. The buffer did not contain MgCl_2_ to prevent ATP hydrolysis. Peak fractions of purified complexes were combined and 0.5 μg protein was mixed with buffer to final concentrations of 1 mM ATP and 5 mM MgCl_2_ in a total volume of 40 μl. Kinetic measurements were carried out using 0.25 μg protein combined with buffer resulting in various ATP concentrations. After incubation for 10 min at 37 °C, samples were frozen in liquid nitrogen. Free phosphate released by ATP hydrolysis was determined adding 800 μl 0.9 mM malachite green, 90 mM ammonium molybdate, 1 M hydrochloric acid, 0.1% Triton X-100 and 100 μl 1.8 M citric acid. After incubation at room temperature, 200 μl of the samples were transferred to 96-well plates and absorption at 640 nm was measured with a Synergy H1 Hybrid Reader (BioTek). ATPase activity was calculated after subtraction of routinely included reactions without Pex1/6 complex, using a potassium phosphate calibration curve and Michaelis–Menten plots were determined using GraphPad Prism 5.0 (GraphPad Software).

### Oleate growth assays

Cells expressing wild-type, no or mutated Pex1, Pex6 alleles (Supplementary Table 4) were grown in YPD (1% w/v yeast extract, 2% w/v bacto peptone and 2% w/v glucose) at 28 °C for 16 h and subsequently washed with water. A 10-fold serial dilution starting with 2 × 10^4^ cells was spotted on agar plates containing 0.67% (w/v) yeast nitrogen base (without ammonium sulfate and without amino acids), 0.2% (v/v) oleic acid (Fig. 3b) or 0.1% (v/v) (Supplementary Fig. 5c) oleic acid, 0.5% Tween 20 (v/v), 0.3% (w/v) yeast extract, 0.5% (w/v) ammonium sulfate and suitable amino acids, adjusted to pH 6. Control plates include 0.2% (Fig. 3b) or 2% (Supplementary Fig. 5c) glucose instead of oleic acid. Oleate plates were incubated for 5–6 days and control plates for 3 days at 28 °C.

### Electron microscopy and image processing

Purified wild-type Pex1/6 complexes were diluted to 40–50 μg ml^−1^ in 20 mM Tris-HCl pH 7.5, 20 mM NaCl and 10 mM MgCl_2_ and incubated with ATPγS (2.5 mM) or ATP (5 mM) and Pex1GST/Pex6 complexes with ADP (5 mM) in 20 mM Tris-HCl pH 7.5, 20 mM NaCl and 10 mM MgCl_2_. For the ADP-AlFx state, 2 mM AlCl_3_ and 8 mM NaF^10^ were added to purified wild-type Pex1/6 complexes diluted to concentrations stated above in 20 mM HEPES pH 7.5, 50 mM NaCl, 2 mM MgCl_2_ and 2 mM ADP. In total, 3.5 μl protein solution were applied on glow-discharged 400-Cu mesh continuous carbon grids (Quantifoil) for 45 s, blotted to near dryness and negatively stained with 3.5 μl 2% (w/v) uranyl acetate. Negative staining of adsorbed Pex1WB^ATP^/6, Pex1/6WB^ATP^ and Pex1/6 DWB^ATP^ complexes was performed on four successive drops of 25 μl uranyl acetate. Grids were incubated for 10 s on each drop and subsequently blotted to near dryness, according to (ref. 41). Separated Δ188Pex1/Pex6 complexes were incubated with ATPγS (2.5 mM) and stained accordingly. Images were recorded on a FEI Morgagni electron microscope equipped with a SIS Megaview 1K CCD camera at a nominal magnification of × 60,000. Images for 3D reconstructions were recorded using a Tecnai G2 Spirit transmission electron microscope operating at 120 kV equipped with a 2,048 × 2,048-pixel CCD camera (FEI Company). The micrographs were taken at a nominal magnification of × 103,448 with defocus ranging from 350–1,000 nm and sampled at 2.9 Å per pixel at the specimen level. A total of 3,895 (Pex1/6^ATPγS^), 2,948 (Pex1/6^ATP^), 1,488 (Pex1/6^ADP^), 2,394 (Pex1/6^ADP-AlFx^), 2,064 (Pex1WB^ATP^/6), 2,048 (Pex1/6WB^ATP^), 2,073 (Pex1/6 DWB^ATP^) and 400 (Δ188Pex1/Pex6^ATPγS^) particles were selected either manually using the MRC program Ximdisp^42^ or template-based using FindEM^43^. The defocus and astigmatism of the micrographs were determined using CTFFIND3 (ref. 44) and phase correction was done in SPIDER^45^,^46^. Single-particle analysis was done in IMAGIC-5 (ref. 47). Particle images were normalized, band-pass filtered between 200 and 10 Å and centred by iterative alignments to their rotationally averaged sum. Initial class averages, containing 5–10 images, were obtained by three to seven rounds of classification based on multivariate statistical analysis followed by multi-reference alignment using classes with distinct views as new references. For each data set an initial 3D reconstruction was created by angular reconstitution imposing threefold symmetry and used as a reference for projection matching in SPIDER. After 6–14 rounds of Euler angle assignment by projection matching, ∼90% of the angles were stable (Supplementary Fig. 5e). For symmetry-free Pex1/6^ATPγS^ C1 and Pex1/6^ATP^ C1 reconstructions, particle orientations of respective data sets were determined in two rounds of projection matching in SPIDER using the threefold symmetrized starting model. Subsequently, particle orientations were determined without applying symmetry. After 14 (Pex1/6^ATPγS^ C1) or 15 (Pex1/6^ATP^ C1) rounds of projection matching, ∼90% of Euler angles were stable. The resolutions of the final 3D structures (Pex1/6^ATPγS^ 21 Å, Pex1/6^ADP-AlFx^ 23 Å, Pex1/6^ATP^ 23 Å, Pex1/6^ADP^ 24 Å, Pex1/6 DWB^ATP^ 23 Å, Pex1/6WB^ATP^ 23 Å, Pex1WB^ATP^/6 23 Å, Pex1/6^ATPγS^ C1 23 Å and Pex1/6^ATP^ C1 25 Å; Supplementary Fig. 2c) are estimated by Fourier Shell Correlation with a 0.5 correlation cutoff.

### Protein sequence analysis

Multiple protein sequences are aligned using the ClustalW algorithm^48^,^49^ and output files are visualized and edited using Jalview 2.7 (ref. 50).

### Atomic structure fitting

Homology models for Pex1 and Pex6 D1/D2 domains were created using the HHpred server^51^ followed by model building using MODELLER^52^, based on known p97 crystal structures (pdb-ID: 3CF2, 3HU3, 3CF0). p97 (pdb-ID: 3CF3) was automatically fitted into the Pex1/6^ATPγS^ EM density map using ‘Fit in Map', implemented in the USCF Chimera package^53^. Homology models of Pex1/Pex6 D1 or D2 AAA+ domains are automatically fitted and optimized based on the best cross-correlation using ‘Fit in Map'. All starting positions tested resulted in the same local optima of the models in the EM density map, indicating only one local minimum for each fit. Fitting of p97 D2 domains to our Pex1/6^ATPγS^ and Pex1/6^ADP-AlFx^ EM reconstructions or swapping Pex1 or Pex6 D1/D2 domains matches the fit of Pex1/6 homology models, indicating that the overall shape of the AAA+ domain establishes the fits. Fitted Pex1 or Pex6 AAA+ domains are symmetrized using ‘pdbsymm' in Situs^54^. All 3D reconstructions are aligned to the ATPγS-bound map in Chimera. Map segmentation was done in Chimera. Figures are prepared using Pymol (www.pymol.org) and Chimera. In case of symmetry-free EM density maps, homology models of Pex1/6 ATPase domains were fitted automatically to each D2 domain individually. Figures were prepared using Pymol (www.pymol.org) and Chimera.

## Additional information

**Accession numbers**: The negative stain maps have been deposited to the EM Data Bank (http://www.emdatabank.org/) with accession codes EMD-2583 (Pex1/6^ATPγS^), EMD-2584 (Pex1/6^ADP-AlFx^), EMD-2585 (Pex1/6^ATP^), EMD-2582 (Pex1/6^ADP^), EMD-2588 (Pex1/6 DWB^ATP^), EMD-2586 (Pex1/6WB^ATP^) and EMD-2587 (Pex1WB^ATP^/6).

**How to cite this article**: Ciniawsky, S. *et al*. Molecular snapshots of the Pex1/6 AAA+ complex in action. *Nat. Commun*. 6:7331 doi: 10.1038/ncomms8331 (2015).

## Supplementary Material

Supplementary Information Supplementary Figures 1-5, Supplementary Tables 1-4, and Supplementary References

Supplementary Movie 1 Morph between 3D reconstructions of the Pex1/6 DWBATP and Pex1WB/6ATP
